# Buffalo Medical and Surgical Association

**Published:** 1882-10

**Authors:** Clayton M. Daniels

**Affiliations:** Secretary


### Society Reports.

### Buffalo Medical and Surgical Association.

*Regular Meeting, September 4th, 1882.*

President, Dr. A. R. Davidson, in the Chair. Dr. C. M. Daniels, Secretary.

Present—Drs. Cronyn, Barrett, Bartlett, Storck, Abbott, McBeth, Fowler, Gay, Burghardt, Runner, Rochester, Cary, Brecht, Stockton, and by invitation, Drs. F. H. Potter, Frank, Fell, Clark, and Mr. J. W. Ward.

*Essay*—By Dr. George E. Fell, subject: "The Microscope and Medicine." The Essayist stated that our medical schools, or the great majority of them at least, do not profess to teach anything regarding the technology of microscopic investigation. Practitioners are sent out into the world, *suitably qualified* it *may* be, to practice medicine, but not knowing the difference between an eye-piece and an objective; totally unfit, so far as their medical education qualifies them, to use the microscope in the examination of the simplest object; knowing veritably nothing regarding the instrument which has done more to advance the science of medicine than any other in the long category of instrumental accessories. While reasonable persons will admit that "knowledge is power," at least "in the long run," it appears that many of our leading medical schools are controlled by the sentiment that microscopical knowledge does not enhance the physician's skill. Without a knowledge of histology, pathology, the normal and abnormal secretions of the body as revealed by the microscope, he is as fully—yes, according to many—better qualified to practice medicine than if he possessed this knowledge. That such a condition of things is to be deplored no thoughtful practitioner will deny. It would appear that the revelations already made by this wonderful instrument, the progress in medical science credited to its use, would have produced a different condition of affairs. It will be generally conceded that the value of the microscope is directly proportionate to the experience of the practitioner in its use, and a knowledge of histology and pathology is virtually necessary to its intelligent application in medicine. On this account the want of early training in these branches unquestionably deters many of the active practitioners of the day from giving any attention to those subjects. In the midst of an active practice it is quite improbable that time will be found to take up a new occupation, and, as considered by many of the older physicians, one of questionable value. The writer considered that the medical colleges were largely responsible for professional ignorance on this subject, and quoted Prof. Chas. H. Stowell as saying:

"I hold the great majority of the medical colleges of this country directly responsible for the lack of this love for scientific research. They seek to please the student by prejudicing him against the scientific investigation of disease, and they become practitioners before they are aware of their ignorance of matters that should have been familiar to them during the term of their pupilage. * * * But to the coming physician must we, as microscopists, look for a solution of the more exact nature of disease and the more effective methods for its prevention and cure." He spoke at length and *con amore* of the great value ot the microscope in clinical medicine and briefly referred to the brilliant discoveries of Pasteur, Koch, Beale, and others, which the microscope alone had made possible.

*Discussion*—Dr. Cronyn had listened to the paper with pleasure; it did not, however, open an opportunity for much discussion. The views so well advanced would, he thought, meet the approval of all present. Although of late years the demands of a large practice had not given him the time to devote that personal attention to the use of the microscope which he should like, he considered it of the greatest practical value and believed that microscopy being taught practically in the medical schools would meet a needed want. He related several cases in which its value as an aid to diagnosis was signally displayed.

Dr. Barrett considered the subject a difficult one to discuss, as he considered the microscope the greatest instrument for the furtherance of knowledge, comparing it as great in its revelations of minuteness as the telescope in greatness, and that in medicine it was now invaluable as it so clearly defined function. He thought its future would be more in the hands of specialists, as the busy practitioner could not be expected to become an expert microscopist.

Dr. Rochester was sorry the essayist had made such sweeping assertions regarding the want of knowledge of the microscope among physicians in general, as he could hardly believe that the facts would warrant it. He thought, too, that the medical colleges devoted more attention to microscopy than was intimated by the essayist. In the Buffalo Medical College, courses had been established and the student could get a very good practical knowledge of the microscope. Students, however, did not seem inclined to embrace the advantages presented. He also called attention to the fact that as yet great diversities of opinion may be found among different microscopists, showing a lack of certainty among those who were looked to as our authorities. He recommended that at least a general knowledge of the instrument should be had among physicians and that specialists should be encouraged.

Dr. Bartlett thought that the younger members of the profession had greater opportunities and should be the ones to advance microscopic knowledge. The great field seemed to be to find the source or germ of diseases of a special character, and he believed we should soon know the real cause of zymotic diseases.

Mr. Ward believed the physicians were really more interested than the essayist gave them credit for. Without practice it is very difficult to see all that the microscope wants to tell, and that disputes often arose among those best fitted to judge. In general the physician best appreciates its use. He had the satisfaction once to observe living trichinæ moving in flesh. Patient was supposed to have died from typhoid fever, the microscope alone cleared up the doubt. He thought the microscope would naturally come into the hands of physicians.

Dr. Davidson was of the opinion that Dr. Fell had somewhat exaggerated the value of the microscope in medicine; although its usefulness was conceded as an important adjunct to other means of diagnosis, it was questionable whether, in clinical medicine, it was entitled to a much higher rank than the fever thermometer or stethoscope. He did not, however, believe that the essayist had exaggerated the lack of microscopical knowledge on the part of the general profession for which the medical colleges were largely responsible. During later years, at the Buffalo college, as Dr. Rochester had stated, opportunities had been presented to the student to gain some knowledge of microscopy, but the course was accompanied with extra expenses, and the acceptance of it purely optional on the part of the student. He believed that some knowledge of the microscope and of the way to use it should be made obligatory—for with that foundation the young practitioner would be pretty certain to devote a good deal of his leisure during the first years of practice to its study—and thereby make the possession of a microscope of real value to him. His experience had been that those who had not acquired a knowledge of the microscope at college seldom obtained an instrument until they had got into an active practice; very rarely then do they take the time to acquire any correct knowledge of its use, and the possession of it was often injurious in that there was a temptation to guess at what was seen, instead of taking the advice of a qualified observer.

Dr. Fell believed that the study of the microscope should be made obligatory to the student, and that, if it was more fully understood, it would be better appreciated.

*Voluntary Communications*—Dr. Cary related a case of transfusion where there was hemorrhage from leg after compound fracture, it being a general oozing from capillaries, until patient was in a critical condition. Bleeding could only be checked by application of ice. About forty-eight hours before transfusion the hemorrhage apparently ceased and patient seemed dying from exhaustion; was unconscious; no pulse at wrist. Transfusion in ordinary way; about three ounces of blood used, which was followed by immediate improvement; flushing of face, consciousness; comparatively good pulse, and recovery seemed assured. A peculiar condition followed, being a hemorrhage into cellular tissue about the neck, there being found after death, which occurred a few hours later, several sacs filled with blood.

Dr. Barrett mentioned two cases of persistent hemorrhage from extracting teeth, it being capillary and checked with great difficulty.

Dr. Gay exhibited a lady's shawl pin, which he had removed from the male urethra by performing the operation of external urethrotomy. The pin measures five inches in length; the head is three-eighths of an inch in diameter. It was so securely lodged well down in the urethra as to foil all attempts on the part of the patient and his physician to remove it.

Dr. Gay also exhibited some specimens of gall-stones, one which measured an inch in diameter, which had been taken from the gall-bladder at an autopsy, recently made. A few years ago, Dr. G. reported to the association two cases, and exhibited gall-stones from a third case, all three of which he had treated with large doses of olive oil, with ordinary doses of podophylin. The gall-stones found at the autopsy were from the same patient as were those exhibited at a meeting held December 4, 1866. Doubts at that time were expressed about their being gall-stones. A member is reported as saying (*Buffalo Medical and Surgical Journal*, vol. vi, p. 214) that "They are soft and pliable and not hard like gall-stones. * * To say the substances, presented by Dr. Gay, are true biliary calculi, is more than their appearance would warrant. * * They appear to be composed of cholesterine and fœcal matter and probably formed in the intestinal canal." The gall-stones now shown were, when taken from the gall-bladder, of the consistence of old cheese and were easily cut with the knife. After being dried, they have become hard, like stone. As those formerly exhibited were identical with these (except the larger one) in color, shape, size and consistency, the autopsy proves that he was not in error in calling the former gall-stones. The patient did not die in consequence of them, nor had he any symptoms of their presence for the past fifteen years.

Dr. Cronyn related a case where patient was cut by glass, the brachial ulnar and radial arteries all being severed; there being no pulse at wrist. Three accupuncture pins were used with figure of eight sutures. In three days the two lower pins were removed and there was perceptible feeling of radial pulse, evidently showing that the inflammatory action had increased dilatation of the vessels on lateral and posterior aspect of arm.

The question regarding the value of olive oil as a gall-stone solvent was discussed by Drs. Rochester, Gay and Davidson, the prevailing opinion being that it only acted mechanically in facilitating their passage.

Dr. Bartlett asked if any one present knew of anything that would dissolve gall-stones.

Dr. Rochester said that chloroform, given internally, was highly recommended.

*Prevailing Diseases*—Dr. Cronyn reported scarlatina and diarrhœa.

Dr. Bartlett, dysentery and occasionally diphtheria, and related a case where child had a diphtheritic-like eruption externally three days before throat symptoms appeared, and which terminated fatally.

Adjourned.

CLAYTON M. DANIELS, *Secretary.*

